# ‘*In silico* expression analysis’, a novel PathoPlant web tool to identify abiotic and biotic stress conditions associated with specific *cis*-regulatory sequences

**DOI:** 10.1093/database/bau030

**Published:** 2014-04-10

**Authors:** Julio C. Bolívar, Fabian Machens, Yuri Brill, Artyom Romanov, Lorenz Bülow, Reinhard Hehl

**Affiliations:** Institut für Genetik, Technische Universität Braunschweig, Spielmannstr 7, 38106 Braunschweig, Germany

## Abstract

Using bioinformatics, putative *cis*-regulatory sequences can be easily identified using pattern recognition programs on promoters of specific gene sets. The abundance of predicted *cis*-sequences is a major challenge to associate these sequences with a possible function in gene expression regulation. To identify a possible function of the predicted *cis*-sequences, a novel web tool designated ‘*in silico* expression analysis’ was developed that correlates submitted *cis*-sequences with gene expression data from *Arabidopsis thaliana*. The web tool identifies the *A. thaliana* genes harbouring the sequence in a defined promoter region and compares the expression of these genes with microarray data. The result is a hierarchy of abiotic and biotic stress conditions to which these genes are most likely responsive. When testing the performance of the web tool, known *cis*-regulatory sequences were submitted to the ‘*in silico* expression analysis’ resulting in the correct identification of the associated stress conditions. When using a recently identified novel elicitor-responsive sequence, a WT-box (CGACTTTT), the ‘*in silico* expression analysis’ predicts that genes harbouring this sequence in their promoter are most likely *Botrytis cinerea* induced. Consistent with this prediction, the strongest induction of a reporter gene harbouring this sequence in the promoter is observed with *B. cinerea* in transgenic *A. thaliana*.

**Database URL:** http://www.pathoplant.de/expression_analysis.php.

## Introduction

Eukaryotic gene expression is largely regulated by the binding of transcription factors (TFs) to *cis*-sequences in promoter regions. To identify *cis*-sequences that may have a specific regulatory function, pattern recognition programs can be used to identify common *cis*-sequences in promoters of a set of co-regulated genes (1). Such bioinformatic approaches have been used in recent years for the discovery of large numbers of conserved *cis*-regulatory sequences, many of which are not associated with known *cis*-sequences (2–6). The abundance of predicted *cis*-sequences represents a major challenge for defining their function in regulation of gene expression.

One way to identify the conditions upon which a *cis*-sequence may confer gene expression is to analyse if genes harbouring this sequence in their promoter show a specific expression profile under certain environmental conditions (7). This has been done for many sequences predicting known and novel expression profiles (8–10). In several cases, these sequences have also been tested experimentally (8, 11). This shows that such an approach is a useful way to identify the possible function of specific *cis*-sequences. For such predictions, it may be particularly helpful to have an online web tool that permits such an analysis for any given *cis*-sequence. To facilitate such an analysis, a new web tool designated ‘*in silico* expression analysis’ has been implemented in the PathoPlant database (12, 13). The database is manually annotated with data from the literature. Currently, it contains data for 99 plant species and varieties, 107 pathogens and 638 molecules from 619 references (14). These data represent 350 interactions and 370 reactions. Via a recently developed function, molecules and reactions annotated within PathoPlant can be visualized as signalling pathway maps. A map of all reactions and molecules annotated to PathoPlant can be generated as well as specific pathway maps starting from a selected molecule (14). In addition, 144 different microarray data sets from *Arabidopsis thaliana*, corresponding to 36 different abiotic and biotic stimuli, have been annotated to PathoPlant.

The newly developed ‘*in silico* expression analysis’ tool can be used to identify the biotic and abiotic stimuli that may induce or repress expression of genes harbouring a specific *cis*-sequence under investigation. Using the web tool, the potential *cis*-sequence can be submitted to perform a genome-wide *A. thaliana* promoter screening. The gene sets obtained are used to calculate mean induction factors for every *A. thaliana* microarray experiment stored within PathoPlant. A negative ‘induction factor’ would mean that these genes are downregulated. These mean values are normalized according to overall expression values of each stimulus. This results in a ranked list of microarray experiments according to their mean induction factors. The most probable stimuli to which genes harbouring the potential *cis*-element in their promoter are responsive to can be identified by looking at the highest-ranked stimuli for upregulated genes or the lowest ranked stimuli for downregulated genes. This article describes the implementation of the web tool and a proof of concept analysis with known *cis*-sequences that confer stress-responsive gene expression. In addition, using the recently identified WRKY70 TF binding site CGACTTTT (15) and reporter gene technology in transgenic *A. thaliana*, the prediction made by an ‘*in silico* expression analysis’ was confirmed.

## Methods

### Microarray data

The PathoPlant database harbours microarray expression data primarily for biotic and abiotic stress conditions (5, 13). Most of the microarray data were generated in the AtGenExpress project (7) and were downloaded from TAIR, NASCArrays, ArrayExpress and NCBI GEO (16–19). Microarray data were normalized using the Affymetrix MAS5 algorithm (20). Currently, 144 different microarray data sets corresponding to 36 different abiotic and biotic stimuli have been annotated to PathoPlant. All data sets, array type and a link to the expression set used for downloading the data can be found on the documentation page of PathoPlant at http://www.pathoplant.de/. In addition to the 144 data sets for abiotic and biotic stimuli, two data sets correspond to inflorescence-specific gene expression. The data can be accessed through the ‘Microarray expression’ tool at http://www.pathoplant.de/ as described earlier (13).

### The ‘*In silico* expression analysis’ web tool

To bioinformatically assess the functionality of identified *cis*-sequences, a new web tool was developed that can be accessed online at http://www.pathoplant.de/expression_analysis.php. It provides a bioinformatic approach to investigate whether genes harbouring specific *cis*-sequences are responsive to certain biotic and abiotic stresses. The web tool validates the possible role of the *cis*-sequence to confer the identified stress-responsive gene expression. The tool uses microarray expression data from the PathoPlant database to calculate mean induction factors for gene sets that contain a submitted sequence within their promoters. Positive mean induction factors (>1) describe upregulated genes, and negative mean induction factors (<−1) describe downregulated genes. The statistical significance of the mean induction factors is assessed by calculating a *P-*value and by applying a false discovery rate (FDR) *P*-value adjustment. Such information is used to evaluate the probability of a given sequence to be a putatively functional *cis*-element.

After submitting the sequence to the ‘*in silico* expression analysis’ web tool, a genome-wide promoter screening for sequence occurrences is performed. To permit these screenings, all *Arabidopsis* gene promoters were extracted from the TAIR8 genome data files annotated to the AthaMap database (21). Using this information, the transcription start site (TSS) (if known, otherwise the start codon) of all *Arabidopsis* genes was determined as the gene start position to extract the 250-, 500- or 1000-nt region upstream of this position. The default setting is a 500-nt upstream region. This region may be sufficient for promoter analyses as shown by previous *cis*-sequence distribution analyses (22). When choosing 1000 nt as upstream region, a significant number of sequences will also contain a segment of the neighbouring gene because of the high gene density in the *Arabidopsis* genome. All promoter sequences with the three different promoter sizes are stored in FASTA format files in the PathoPlant database containing *Arabidopsis* gene identifiers and the corresponding DNA sequences. These files are then accessed by the in silico tool to find exact matches of the submitted *cis*-sequence in sense and antisense orientations within the gene promoter region selected online (250, 500, 1000 nt). The induction factors of the genes found are retrieved from the database and are used to calculate mean induction factors using microarray expression data for each one of the 146 experiments stored in the PathoPlant database. The average mean expression *Avg*(*w, s*) of a gene set (*w*) under a stress (*s*) is given by the geometrical mean of the induction factors:
(1)
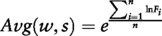

where

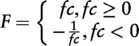

and *fc* denotes the induction factors FOLD_CHANGE value of a given gene from set *w* under stress *s,* while *n* denotes the number of genes in a set *w.* Equation (1) is applied for each microarray experiment, and in this way, expression data for the gene sets under different stresses is retrieved. For comparability among the different experiments, these values are normalized. For this purpose, Equation (1) was also used to calculate the overall means of all genes for each of the different stresses *Avg*(*all, s*). These values constitute normalization factors and were stored in a table that the in silico tool accesses to normalize each newly calculated mean value for the genes identified with the ‘*in silico* expression analysis’ tool. The normalized values *NAvg*(*w, s*) under stress *s* are given by:
(2)
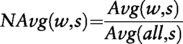

where *Avg*(*w, s*) denotes the average mean expression for a gene set *w* under stress *s* and *Avg*(*all, s*) is the average mean expression of all genes under stress *s*. After normalization, results are ordered according to mean induction factor values, which result in a ranking of experiments.

Statistical significance of the average mean expression values calculated for the different gene sets is assessed by means of a raw *P-*value calculation with a Student's *t*-test and subsequent FDR *P*-value adjustment.

The following data are used for raw *P*-value calculation for a given gene set that harbours the submitted *cis*-sequence within the selected promoter region in comparison with all genes present on the microarray chip: the average mean expression (*mean*), variance of the individual induction factors (*var*) and number of induction factors (*n*) used to calculate expression. These data are determined by the PathoPlant database server each time a new calculation is performed. The raw *P*-value is calculated by the Apache web server using PHP (version 5.3.11) and the stats_cdf_t function from the PECL stats extension package. This function accepts the *t*-value that is calculated from *mean*, *var* and *n* as parameters to return the raw *P-*value as *observed significance* associated with an one-tailed unpaired *t*-test. This compares the distribution of the individual induction factors obtained for the genes harbouring the submitted *cis*-sequence within the selected promoter region under each stress condition with the overall distribution of induction factors for all genes under the same stress condition. Because *t*-test and *P*-value calculation is performed on correlated data sets, Benjamini–Hochberg (BH) FDR *P*-value adjustment is applied to calculate BH-adjusted (FDR) *P*-values (23). By using standard PHP functions, the adjustment is performed from a sorted list of raw *P*-values and the number of data sets (24). These data are added to the output created by the in silico expression analysis tool, and in that way, it is possible to determine the significance value of a calculated average mean expression value for a given stress.

Once mean induction factors, raw and BH-adjusted (FDR) *P-*values are calculated, results are ordered according to mean induction factor values. The results are displayed online, and they can be resorted according to the calculated raw or BH-adjusted (FDR) *P-*values or according to the stimulus.

### T-DNA constructs

The transfer DNA (T-DNA) construct for *A. thaliana* transformation was generated in the vector pGPTV_bar (25). For this, the *cis*-sequence was amplified by polymerase chain reaction (PCR) from a plasmid containing four copies of sequence 20 in pBT10-GUS (5) using primers 5′-TAGCAAGCTTGAATTCGGCGCGCCACTAGT-3′ and 5′-ATCCCGGGGGTGGCCACTCGAGC-3′. The PCR product was cut with HindIII and SmaI and cloned into the T-DNA vector digested with the same enzymes. After sequencing of the recombinant T-DNA vector, the resulting plasmid pSeq20_GPTV_bar was transformed into *Agrobacterium tumefaciens* C58C1 (26). The plasmid pSeq20_ GPTV_bar contains four copies of sequence 20 upstream of a minimal promoter (TATA-box) linked to the *uid*A reporter gene encoding β-glucuronidase (GUS). For all recombinant DNA, work standard protocols were used (27). Sequence analysis was done by GATC Biotech (Konstanz, Germany).

### Transformation of *A. thaliana*

*Arabidopsis thaliana* accession Col-0 was transformed following the floral dip transformation protocol (28). For transformation, *A. tumefaciens* C58C1 harbouring plasmid pSeq20_GPTV_bar was used. After harvesting the seed of the transformed plants, transgenic plants were selected on medium containing 30 mg/l phosphinothricin. A total of 14 independent transformants were obtained. Segregation analysis of transgenic offspring in the T1 generation revealed that five lines harbour one T-DNA locus (lines 3, 6, 8, 13 and 14). These lines were subjected to pathogen infection and reporter gene assays.

### Pathogen infection

All pathogen infections were performed with 5- to 8-week-old transgenic *A. thaliana* lines grown under short-day conditions (8 h light, 16 h dark). For infection with *Botrytis cinerea* (strain B05.10), the fungus was grown on potato dextrose agar (PDA) medium (Carl-Roth, Karlsruhe, Germany) at 25°C in 150-mm petri dishes. Infections were done according to Mengiste *et al.* (29). For infection, spores of a 10-day-old *B. cinerea* culture were recovered using 10–15 ml of Sabouraud maltose broth [(SMB), 40 g/l maltose, 10 g/l pepton, pH 5.6)]. Spores were scrabbed from the *B. cinerea* mycelium with a glass pipette. The spore suspension was recovered by filtration through gauze, and the spore concentration was determined using a haemocytometer. For infection, the suspension was adjusted to 1 × 10^5^ spores/ml in SMB, and a 10-µl droplet of the spore suspension was applied to the *A. thaliana* leaves to be infected. To maintain high humidity, the plants were kept in closed containers saturated with water vapour. After 3 days, leaves were harvested for reporter gene assays.

For infection with *Pseudomonas syringae* pv. *tomato* DC3000, a virulent (containing the vector pVSP61) and an avirulent strain (containing the vector pVSP61 expressing *avrRPM1*) were used. Both strains were grown on king's B (KB) medium (20 g/l pepton, 1.5 g/l K_2_HPO_4_, 1.5 g/l MgSO_4_ 7H_2_O, 10 ml/l glycerol, 15 g/l agar) containing kanamycin (50 mg/l) and rifampicin (50 mg/l). After incubation for 2 days at 25°C, 5 ml of liquid KB medium (with antibiotics) was inoculated with a single colony and grown overnight at 25°C. Subsequently, 50 ml of prewarmed KB medium was inoculated with 1 ml from the over-night culture and incubated for another 5–6 h at 25°C. The cells were precipitated by centrifugation (3000*g*, 10 min) and resuspended in sterile deionized water to a final optical density at 600 nm of 0.2. A 1:10 dilution of this suspension was used for leaf infiltration. For this, a needleless syringe containing 1 ml of the diluted bacterial suspension was used to infiltrate the abaxial side of a leaf by slightly pressing the syringe against the leaf. The infiltrated area was usually 45 mm in diameter. As a control, water-only infiltrations were performed in the same way. The plants were kept in closed containers for 2 days and subsequently subjected to reporter gene assays.

### Reporter gene assays

After pathogen infection on single leaves of *A. thaliana*, single infected and single non-infected or water-inoculated leaves were subjected to quantitative reporter gene (GUS) assays (30). For this, single leaves were homogenized in liquid nitrogen. Two hundred microliters of GUS extraction buffer (50 mM NaPO_4_, pH 7, 10 mM Na_2_EDTA, 0.1% Triton X-100, 0.1% *N*-laurylsarcosine, 10 mM β-mercaptoethanol) was added, mixed, and the cell debris was precipitated by centrifugation (10 min, 16 000 *g*, 4°C). The supernatant was recovered, and the protein concentration was determined according to Bradford (31). The protein concentration was adjusted to 80 µg/ml using GUS extraction buffer. Twenty-five microliters of the diluted protein extract (2 µg) was transferred into a well of a black 96-well microtiter plate. A total of 225 µl of GUS reaction buffer (50 mM NaPO_4_, pH 7.0, 10 mM Na_2_EDTA, 0.1% Triton X-100, 0.1% *N*-laurylsarcosine, 10 mM β-mercaptoethanol, 1 mM 4-methylumbelliferyl-β-*D*-glucuronide) was added, and the plate was inserted into a TriStar LB 941 microplate reader (Berthold Technologies GmbH & Co. KG, Bad Wildbad, Germany) and incubated at 37°C for 10 min before measurements at 37°C. For continuous measurement of GUS activity, the samples in each well were then measured every 15 min for 1 s for the next 3 h (excitation 360 nm, emission 460 nm). Afterwards, for each well, a linear regression for the time period with a linear increase of fluorescence was performed. Non-linear parts were excluded from the regression. The slope of the regression line was then transformed into pmol 4-MU min^−^^1 ^mg^−^^1^. For this, a calibration of fluorescence units with defined amounts of 4-MU was performed in the TriStar. The results shown here are mean values from two (*B. cinerea*) or three (*P. syringae* strains) independent experiments with two replicates each. Error bars represent standard deviations. The induction factors are calculated from the mean values mentioned above.

## Results

### The ‘*In silico* expression analysis’ online web tool

**Figure 1.**
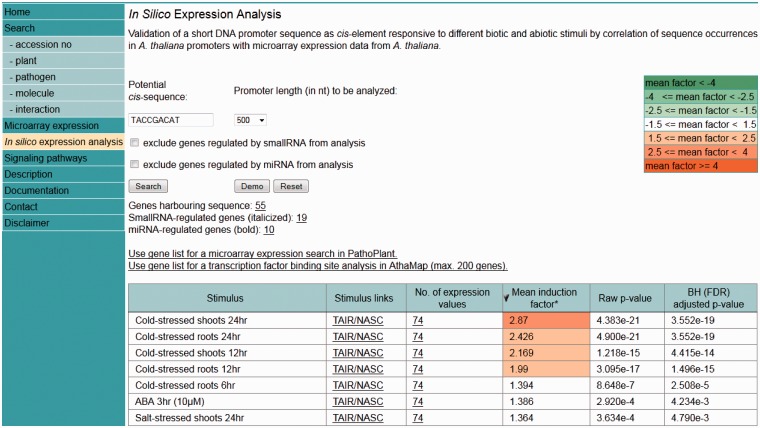
Screenshot of the *‘in silico* expression analysis’ web tool showing the result obtained with the ‘Demo’ sequence.

**Figure 2.**
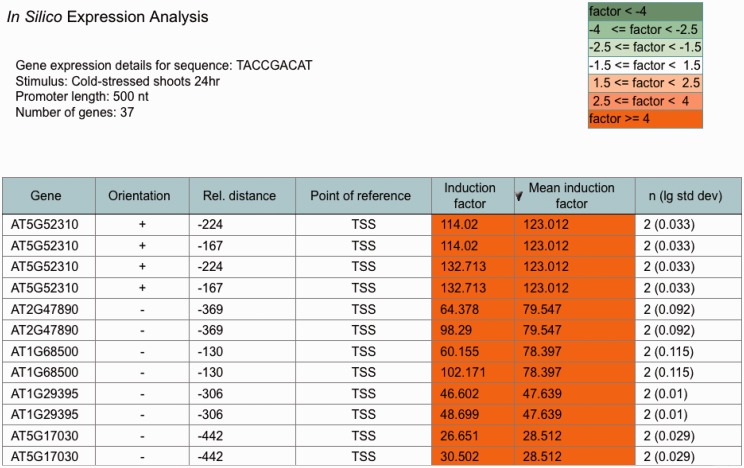
Partial screenshot showing the most highly cold-induced genes identified with the ‘Demo’ sequence. The table identifies the individual genes obtained in the ‘*in silico* expression analysis’ for the selected sequence and the selected stress. Furthermore, it shows the orientation and relative distance of the sequence to the point of reference (TSS) in each gene. The induction factor of each replicate, the mean induction factor and the number of replicates (*n*) is displayed. The table is sorted according to mean induction factor.

An ‘*in silico* expression analysis’ can be performed online to validate short DNA sequences as *cis*-regulatory sequences potentially conferring gene expression to specific biotic and abiotic stress conditions. Figure 1 shows a screen shot of the online tool with the result obtained with the ‘Demo’ sequence. When selecting ‘Demo’, the sequence TACCGACAT appears as the input sequence. This sequence was first identified in the promoter of the drought-responsive *RD29A* gene from *A. thaliana* and was demonstrated to function as a *cis*-acting element involved in the induction of *RD29A* expression by low-temperature stress (32). The *cis*-sequence submitted is used by the web tool to perform a genome-wide *A. thaliana* promoter screening. A 250-, 500- (default) or a 1000-nt-long upstream region can be selected for analysis. There is an option to exclude genes potentially regulated by small RNAs or microRNAs (miRNAs) from the analysis. These were identified in the *A. thaliana* genome and annotated to the AthaMap database, which is linked to PathoPlant (13, 33, 34). In all, 55 genes harbour the ‘Demo’ sequence in the selected 500-nt upstream region (Figure 1). The gene set harbouring the submitted *cis*-sequence in the selected promoter region is used to calculate mean induction factors for every *A. thaliana* microarray experiment stored within the PathoPlant database. These mean values are normalized according to overall expression values of each stimulus. This results in a ranked list of microarray experiments according to their mean induction factors. Consistent with its known function, the result with the ‘Demo’ sequence reveals this sequence to be associated with genes responding to cold stress (Figure 1). The mean induction factor of the genes harbouring this sequence in the selected promoter region is 2.87 for the microarray ‘cold-stressed shoots 24 hr’, followed by four other microarrays for which lower mean induction factors of the genes were determined under cold stress. For each stimulus, the number of expression values used for mean induction factor calculation is given. The corresponding *P-*value is also calculated for each mean factor to assess its significance. The number of expression values, here 74, is linked to a new window. Figure 2 shows a partial screen shot of this window revealing that 37 (‘number of genes’) of the 55 genes are actually on the microarray ‘Cold-stressed shoots 24 hr’ (‘stimulus’). Further information given on this page includes the gene identifier (‘Gene’), the orientation and position of the *cis*-sequence relative to the gene start and the induction factor of each experiment, as well as the mean induction factor of the gene (Figure 2). The orientation and relative distance refers to the distance of the first match position to the point of reference that can either be TSS, if known, or the translation start codon, if the TSS is unknown. The individual and mean induction factors of each gene, as well as the number of replicates (*n*) and the base-10 logarithm of the standard deviation for mean induction factor calculation of each gene, are shown. By default, the result tables are ranked by mean induction factors (Figures 1 and 2) and can be resorted in descending or ascending order by selecting the headers of the tables. By selecting the number of genes (55, Figure 1), a gene list is shown in a table (not shown). In this gene list, gene descriptions are displayed when selecting the arrow next to the table header ‘Gene’. The list of genes obtained by the ‘*in silico* expression analysis’ can be submitted directly to the ‘Microarray expression’ function of PathoPlant to obtain expression data of these genes for all stimuli. In addition, this list can also be transferred to AthaMap’s gene analysis function for a TF binding site analysis (35).

### Validation of the ‘*in silico* expression analysis’ web tool with known *cis*-regulatory sequences

**Figure 3.**
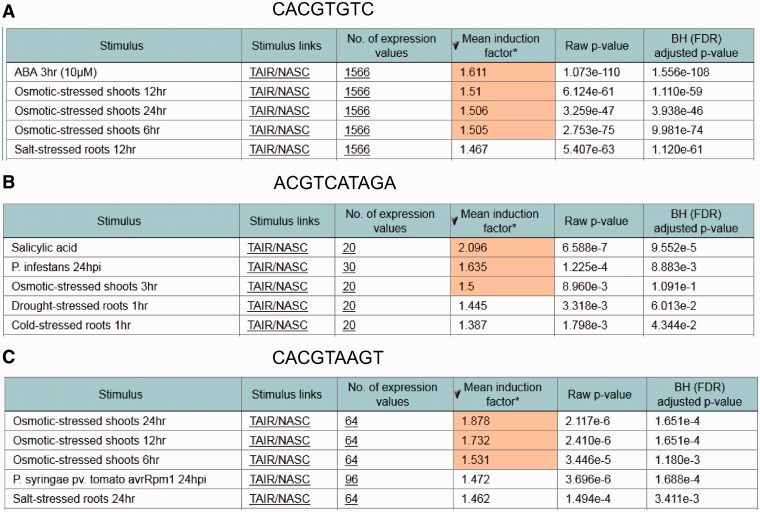
Examples for identifying stress responsive *cis*-elements using the *in silico* expression analysis web tool. In each case, the *cis*-sequence used for *in silico* expression analysis with default settings is shown together with the five microarray expression data sets for which the most significant correlation between occurrence of the *cis*-sequence within the promoter and the expression of the associated genes was detected. *Cis*-sequences are shown. (**A**) An abscisic acid response element. (**B**) A salicylic acid response element. (**C**) A dehydration and senescence response element.

To test the performance of the ‘*in silico* expression analysis’ web tool, additional *cis*-sequences associated with stress-specific gene expression were investigated in addition to the ‘Demo’ sequence used in Figure 1. Figure 3 shows the *cis*-sequences and their predicted stress responsiveness. When the sequence CACGTGTC is submitted using the ‘*in silico* expression analysis’ web tool with default settings, the genes harbouring this sequence within their promoter were found to be most strongly upregulated in the microarray expression data set abscisic acid (Figure 3A). This sequence has previously been associated with abscisic acid-responsive genes (2, 9).

The sequence ACGTCATAGA was previously associated with salicylic acid-responsive genes (36). This sequence is part of LS7, a regulatory sequence from the pathogenesis-related gene *PR1*, which is upregulated by salicylic acid. Figure 3B shows that the microarray expression data set in which genes harbouring this sequence in their promoter were found to be upregulated most strongly is salicylic acid.

Another example is the dehydration and senescence-responsive sequence CACGTAAGT from the *SAG113* promoter (37). Figure 3C shows that genes harbouring this sequence in their promoter were found to be upregulated most strongly by osmotic stress, which causes dehydration.

These examples show that previously identified *cis*-sequences associated with specific stress conditions can be used in the ‘*in silico* expression analysis’ to identify the correct corresponding microarray data sets.

### Prediction of stress conditions for genes harbouring a novel *cis*-regulatory sequence

Recently, a novel elicitor-responsive *cis*-regulatory sequence from *A. thaliana*, CGACTTTT, was predicted bioinformatically and was shown to be a binding site for the WRKY70 TF (5, 15). Although the *cis*-sequence, designated WT-box and bound by WRKY70, is enriched in promoters of genes upregulated in a WRKY70 overexpression line, the primary stimulus to which genes harbouring this sequence in their promoter respond to was unknown.

**Figure 4.**
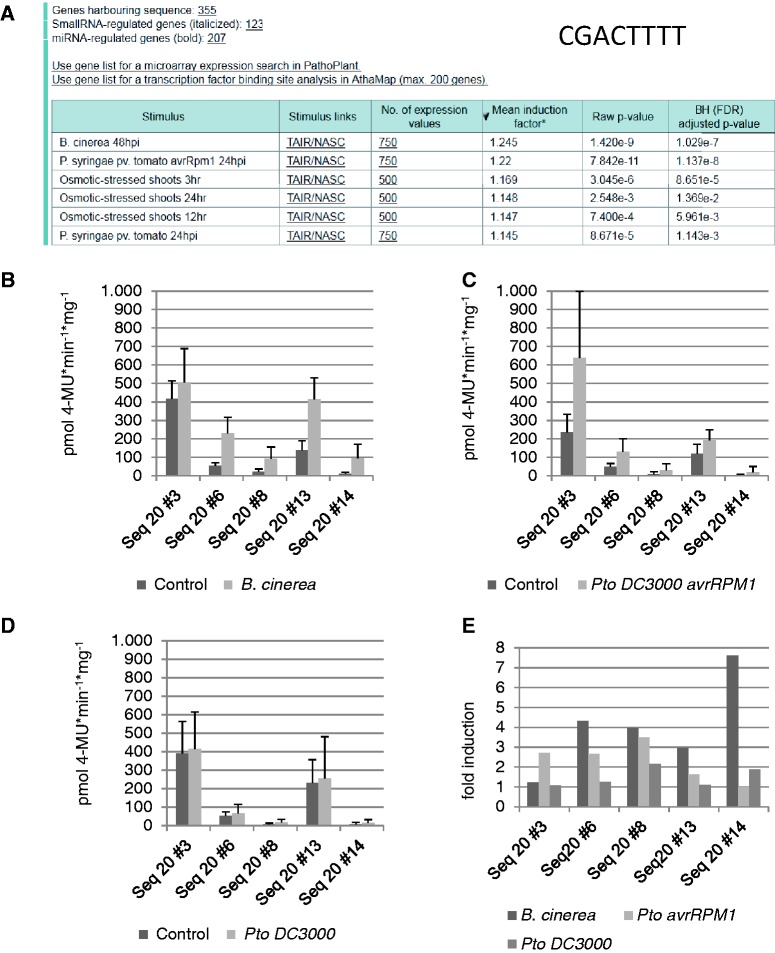
‘*In silico* expression analysis’ and experimental validation of the *cis*-regulatory sequence CGACTTTT. (**A**) The *in silico* expression analysis result with sequence CGACTTTT. (**B–D**) Quantitative GUS expression (pmol 4-MU min^−1 ^mg^−1^) after infection of transgenic *A. thaliana* lines with *B. cinerea* (B), *P. syringae* pv*. tomato* avrRPM1 (C) and *P. syringae* pv. *tomato* (D) compared with the uninfected control. (**E**) The fold induction determined from the change between the GUS values of uninfected and infected plants.

To identify the pathogenic stimulus or any other stress condition that most likely induces genes harbouring the new *cis*-sequence CGACTTTT, an ‘*in silico* expression analysis’ was performed. The analysis was done by submitting the sequence CGACTTTT using default settings of the web tool, except for the small RNA-regulated genes. These were excluded from the analysis because these genes are most likely also post-transcriptionally regulated. Figure 4A shows that 355 genes harbour the *cis*-sequence within a 500-nt upstream region. ‘*In silico* expression analysis’ indicates that genes harbouring this sequence in their promoter are most likely responsive to *B. cinerea* (Figure 4A). The mean induction factor for the genes on the microarray data set was 1.245 (*B. cinerea*), which is low, but the low *P*-value (1.4E-9) for *B. cinerea* may indicate a significant correlation of these genes with their induction by *B. cinerea*.

### Experimental verification of predicted stress conditions for the *cis*-regulatory sequence CGACTTTT

To test the predictions of the ‘*in silico* expression analysis’ experimentally, five independent transgenic promoter-reporter gene lines were analysed. These lines harbour four copies of the *cis*-sequence, containing the sequence CGACTTTT upstream of a minimal promoter and the *uidA* (GUS) reporter gene (Methods).

When these transgenic lines are subjected to infection with spores of *B. cinerea*, all lines show upregulation of the reporter gene compared with the uninfected control (Figure 4B). When the same transgenic lines are subjected to infiltration with *P. syringae* pv. *tomato avrRpm1*, these lines also show upregulation of the reporter gene compared with the uninfected control (Figure 4C). When the same transgenic lines are subjected to infiltration with *P. syringae* pv. *tomato*, these lines do not show upregulation of the reporter gene compared with the uninfected control (Figure 4D). As a negative control, a transgenic line containing only the minimal promoter without the *cis*-sequence upstream of the *uidA* reporter gene was also tested with and without pathogen infection. Reporter gene expression values were always <15 pmol 4-MU min^−^^1 ^mg^−^^1^ (not shown).

When comparing the induction factors of transgenic lines obtained with the three different pathogens, four of the five lines correspond to the prediction made by the ‘*in silico* expression analysis’ because induction is strongest (3–7.6-fold) after *B. cinerea* infection (lines 6, 8, 13 and 14, Figure 4E). In contrast, lines 6, 8 and 13 show a lower mean induction (1.6–3.5-fold) by *P. syringae* pv. *tomato avrRpm1*. The induction factor for *P. syringae* pv. *tomato* is mostly around 1, which means that no reporter gene induction can be detected.

## Discussion

Using pattern recognition programs on promoter sequences of specific gene groups, it is fairly straightforward to identify conserved sequence motifs (1). In contrast to this, the functional analysis of these *cis*-sequences is more elaborate. If conserved sequence motifs were established from co-regulated gene groups, normally the conditions under which the genes are co-regulated are known and may be used to assess whether the sequences identified in the promoters of these genes confer corresponding reporter gene activity (5). However, often only the genomic distribution of short sequences was established, and the functional significance of specific sequences was mainly deduced from known *cis*-sequences (22). In such a study, 65 536 different 8-nt-long sequences, so-called words, were investigated with respect to frequency and positional distribution in the *A. thaliana* genome (9). This study classified the 8-nt-long sequences using gene expression information. Therefore, *cis*-sequences that occur in promoters were associated with specific gene expression profiles. For example, the abscisic acid response element, CACGTGTC, when found in a 1000-bp upstream region strongly correlated with induction by 10 µM abscisic acid (9). This sequence was used as one of the ‘proof of concept’ sequences for the ‘*in silico* expression analysis’ web tool showing the previously determined association with abscisic acid-response genes (Figure 3A). This demonstrates that the ‘*in silico* expression analysis’ web tool, which permits the submission of any putative *cis*-sequence for analysis with respect to gene expression data, will be useful. Although several known *cis*-sequences that were known to be associated with specific expression profiles were correctly identified with the associated microarray data set, the tool has certain limitations. If a *cis*-sequence is too short, the number of genes harbouring the sequence in the promoter will exceed the capacity of the system. If such a sequence is submitted, the web tool will display the statement: ‘The number of genes containing the sequence is too high. Please try a larger sequence or a shorter promoter length’. A more general problem when submitting a *cis*-sequence will be that no stress conditions are identified with the microarrays in the database. This is reminiscent of an earlier analysis in which no stress conditions could be associated with specific *cis*-sequences, although the *cis*-sequences were overrepresented in promoters of genes upregulated in a specific microarray data set (8). This may be due to combinatorial control of gene expression, requiring a second *cis*-sequence for specifying a specific gene expression profile (38, 39). Such combinatorial control of gene expression is known for many *cis*-sequences and their binding TF (40, 41).

In the work presented here, the ‘*in silico* expression analysis’ web tool was successfully used to determine the biotic stress response conditions for genes harbouring a novel *cis*-regulatory sequence designated WT-box (15). This sequence, CGACTTTT, was detected when pattern recognition programs were used on promoters of *A. thaliana* genes upregulated by pathogenic stimuli (5). The reverse complement sequence AAAAGTC was previously detected to be enriched in promoters of genes responsive to flagellin 22, NPP1 and *P. infestans* (4). The sequence CGACTTTT was shown to be bound by WRKY70, extending the range of known WRKY binding sites (15, 42). The ‘*in silico* expression analysis’ performed with this sequence predicted that genes harbouring this sequence are most likely upregulated by *B. cinerea* (Figure 4A). Consistent with this proposal, four of five transgenic *A. thaliana* lines harbouring a reporter gene construct with synthetic promoters containing four copies of this sequence show the most prominent induction after *B. cinerea* infection (Figure 4B). This may indicate that genes harbouring this *cis*-sequence in their promoter may play a role during *B. cinerea* infection. Because this sequence is bound by WRKY70, it is interesting that a *wrky70* mutant is more sensitive to *B. cinerea* infection (43). This may indicate that *B. cinerea*-responsive genes are no longer upregulated in the mutant.

In summary, the work presented here shows that the ‘*in silico* expression analysis’ web tool can be used to predict stress conditions that are most likely inducing genes harbouring a specific *cis*-sequence in their promoter region.
